# Draft Genome Sequences of *Lactobacillus animalis* Strain P38 and *Lactobacillus reuteri* Strain P43 Isolated from Chicken Cecum

**DOI:** 10.1128/genomeA.01229-16

**Published:** 2016-11-03

**Authors:** Morvarid Rezvani, Mary Mendoza, Matthew D. Koci, Caitlyn Daron, Josh Levy, Hosni M. Hassan

**Affiliations:** a Prestage Department of Poultry Science, North Carolina State University, Raleigh, North Carolina; b Microbiology Graduate Program, North Carolina State University, Raleigh, North Carolina; c RTI International, Durham, North Carolina

## Abstract

Here, we present the genome sequence of *Lactobacillus animalis* strain P38 and *Lactobacillus reuteri* strain P43, both isolated from the cecum content of a 4-week old chicken fed a diet supplemented with the prebiotic β(1-4)galacto-oligosaccharide (GOS). These indigenous *Lactobacillus* isolates are potential probiotic organisms for poultry.

## GENOME ANNOUNCEMENT

Members of the genus *Lactobacillus* belong to the lactic acid bacteria (LAB) group. The genus *Lactobacillus* is one of the largest in the LAB group (1). *Lactobacillus animalis* and *Lactobacillus reuteri* have previously been isolated from food and animals (2–6).

Here, we report the genome sequences of *L. animalis* P38 and *L. reuteri* P43 isolated from the cecal microbiota of 4-week old female commercial white leghorn (W-36, Hy-line North America, Mansfield, GA). The birds were housed in climate-controlled HEPA-filtered isolation units (934-1 WP from Federal Designs, Inc., Comer, GA). Water and feed were provided *ad libitum*. Feed consisted of a standard corn-soybean starter diet (NC State Feed Mill) containing 1% commercial galacto-oligosaccharide powder (GOS-55%) (Yakult Pharmaceutical, Tokyo, Japan). The birds were maintained and euthanized according to a protocol approved by the Institutional Animal Care and Use Committee (OLAW#A3331-01). The cecal content from bird #365 was enriched anaerobically in a Coy anaerobic chamber (H_2_ 10%, CO_2_ 5%, and N_2_ 85%) (Coy Lab Products, Grass Lake, MI). The inoculum was enriched two-times using a modified glucose-free MRS (mMRS) medium containing 1.5% agar and 0.5% purified (90%) GOS (gift from Jose Barcena-Bruno). The isolates were selected based on their morphology and physiological properties. They were Gram-positive, rod-shaped, and nonspore formers; catalase negative; grown on glucose and lactose and produced acid and acid-clotted skim-milk. 16S rRNA gene sequencing showed that they were >97% and 95% close to *L. animalis* and *L. reuteri*, respectively. DNA was extracted from cells grown anaerobically in MRS media, using the Promega Wizard genomic DNA purification kit (Promega Corporation, Madison, WI).

Paired-end libraries were created for strains P38 and P43 with an average insert size of 251 bp. Libraries were sequenced on an Illumina MiSeq (Illumina, San Diego, CA) at Argonne National Laboratory (Lemont, IL). Modal k-mer coverage was 1100× for strain P38 and 2200× for strain P43. After error correction, reads were assembled using MIRA v4.9.5 (open source: http://genome.cshlp.org/content/14/6/1147.full). The final reported coverage was 90× for strain P38 and 60× for strain P43. After assembly, contigs with less than 20× coverage or length of less than 200 bp were discarded. The length of the draft genomes of *L. animalis* strain P38 and *L. reuteri* strain P43 are 2,151,063 bp and 1,940,664 bp with G+C contents of 41.1% and 38.7%, respectively.

Phylogenetic trees were built for each strain using other *lactobacillus* genomes for comparison (7). The results placed each strain closest to its presumed species: strain P38 with *L. animalis* and strain P43 with *L. reuteri*, giving evidence that the two strains were indeed the expected species and that the assembly was high quality. In addition, we assessed assembly quality by comparing known metabolisms of each strain to both hand- and RAST-annotated functionality (7).

The draft genomes were annotated using the NCBI Prokaryotic Genome Annotation Pipeline (http://www.ncbi.nlm.nih.gov/genome/annotation_prok/).

### Accession number(s).

The genome sequences of *L. animalis* P38 and *L. reuteri* P43 were deposited in GenBank with accession numbers MCNR00000000 and MCNS00000000, respectively.
